# First-principles study of the complex magnetism in Fe_16_N_2_

**DOI:** 10.1038/s41598-019-44799-8

**Published:** 2019-06-10

**Authors:** Satadeep Bhattacharjee, Seung-Cheol Lee

**Affiliations:** 1 Indo-Korea Science and Technology Center (IKST), Bangalore, India; 2 Electronic Materials Research Center, Korea Institute of Science & Technology, Seoul, Korea

**Keywords:** Atomistic models, Electronic structure, Electronic devices

## Abstract

Magnetic exchange interactions in pure and vanadium (V)-doped Fe_16_N_2_ are studied within the framework of density functional theory (DFT). The Curie temperatures were obtained via both mean field approximation (MFA) and Monte Carlo (MC) calculations based on interactions that were obtained through DFT. The Curie temperature (T_*C*_) for pure Fe_16_N_2_ that was obtained under MFA is substantially larger than the experimental value, suggesting the importance of thermal fluctuations. At zero field, the calculated magnetic susceptibility shows a sharp peak at T = T_C_ that corresponds to the presence of localized d-states. From the nature of the exchange interactions, we have determined the reason for the occurrence of the giant magnetic moment in this material, which remained a mystery for decades. Finally, we posit that Fe_16_N_2_ can also act as a satisfactory spin injector for III–V semiconductors, in addition to its application as a permanent magnet, since it has very high spin polarization (compared to elemental ferromagnets) and smaller lattice mismatch (compared to half-metallic Heusler alloys) with conventional III–V semiconductors such as GaAs and InGaAs. We demonstrate this application in the case of Fe_16_N_2_(001)/InGaAs(001) hetero-structures, which exhibit substantial spin polarization in the semiconductor (InGaAs) region. PACS number: 82.65.My, 82.20.Pm, 82.30.Lp, 82.65.Jv.

## Introduction

Due to their importance in the field of energy-saving technologies for the next generation of electrical devices, permanent magnets that do not contain rare-earths or platinum are a current focus of research^1^. *α*″-Fe_16_N_2_, which is a martensite, has been studied over many years, and several groups have claimed to observe giant magnetic saturation moments in it^2–4^. It was first experimentally prepared by K. H. Jack^5^ during low-temperature annealing of *α*′-FeN. It appeared as a metastable phase that survived up to approximately 500 K, above which it decomposed into *α*-Fe and Fe_4_N.

The existence of a giant saturation moment in *α*″-Fe_16_N_2_ was first reported by Kim *et al*.^2^ and later confirmed by Sugita *et al*.^3^ in single-crystal Fe_16_N_2_ that was grown epitaxially on InGaAs. Since then, several investigations have explored the validation of the giant saturation moment in this material. However, these investigations resulted in more controversy rather than bringing the issue to a firm conclusion as some of these studies confirmed the existence of a giant moment^6,7^ while others were unable to reproduce such results^8^.

Therefore, research on Fe_16_N_2_ has always faced two challenges: (1) stabilizing the material at higher temperature so that it does not decompose into *α*-Fe and Fe_4_N and (2) understanding its complex magnetic behavior, which made the material a “*40 year old mystery*”^9^ among the magnetic materials. In a recent work, we have demonstrated that^10^, Fe_16_N_2_ can be stabilized via vanadium (V) doping. Recently. Ke *et al*.^11^ studied the exchange interaction and ferromagnetic transition temperature for Fe_16_N_2_ and Co- and Ti-doped Fe_16_N_2_ using a first-principles-based method. They obtained the Curie temperature (T_*C*_) via both mean field approximation (MFA) and random phase approximation (RPA). Direct measurement of T_*C*_ of Fe_16_N_2_ is limited due to the decomposition of the material into Fe_4_ and Fe at temperatures above 500 C^2,5^. The Curie temperatures that were obtained theoretically by Ke *et al*. were almost three times higher than the temperature that was obtained by Sugita *et al*. by extrapolating from experimental data that were obtained at lower temperature.

Understanding the magnetism and the exchange mechanism in Fe_16_N_2_ is extremely important. In this paper, we have studied the magnetic exchange interactions and the Curie temperatures in Fe_16_N_2_ and V-doped Fe_16_N_2_. The magnetic exchange constants were obtained via a first-principles-based method and the Curie temperatures were calculated via both MFA and MC simulation. In this paper, we also address the long-standing question about the appearance of anomalously large magnetic moments. We organize the remainder of our paper as follows: In Section II, we discuss the magnetic exchange interactions and the Curie temperatures. In the following Section III, we discuss the possible origin of the giant magnetic moments in the system. We also introduce another potential application of this material in Section IV: Fe_16_N_2_ could be used as a spin injector, in addition to its use as a permanent magnet. Finally, in Section V and VI respectively, our conclusions and the computational methods are presented.

## Results and Discussion

Fe_16_N_2_ has a body-centered tetragonal structure with space group I_4_/*mmm* (number 139). The unit cell contains two N atoms and sixteen Fe atoms. The three Wyckoff positions that are occupied by the Fe atoms are 8h (eight atoms), 4e (four atoms), and 4d (four atoms). Our PBE- optimized lattice constants, namely, a = b = 5.68 Å, c = 6.22 Å, and position parameters, namely, x_8*h*_ = 0.243 and z_4*e*_ = 0.294, well agree with the values that were measured by Jack *et al*.^5^ (a = b = 5.72 Å and c = 6.29 Å, x_8*h*_ = 0.242, and z_4*e*_ = 0.293). For comparison, we have considered two additional variations of PBE, namely, PBE-sol^12^ and revised PBE^13^, which yielded similar results: The average magnetic moments per Fe are 2.43 *μ*_*B*_ (PBE), 2.45 *μ*_*B*_ (PBE-sol) and 2.48 *μ*_*B*_ (rev PBE). As the magnetic moments only differ in the second decimal place, we performed other calculations with the PBE functional only.

### Exchange constants and curie temperature

**Figure 1:**
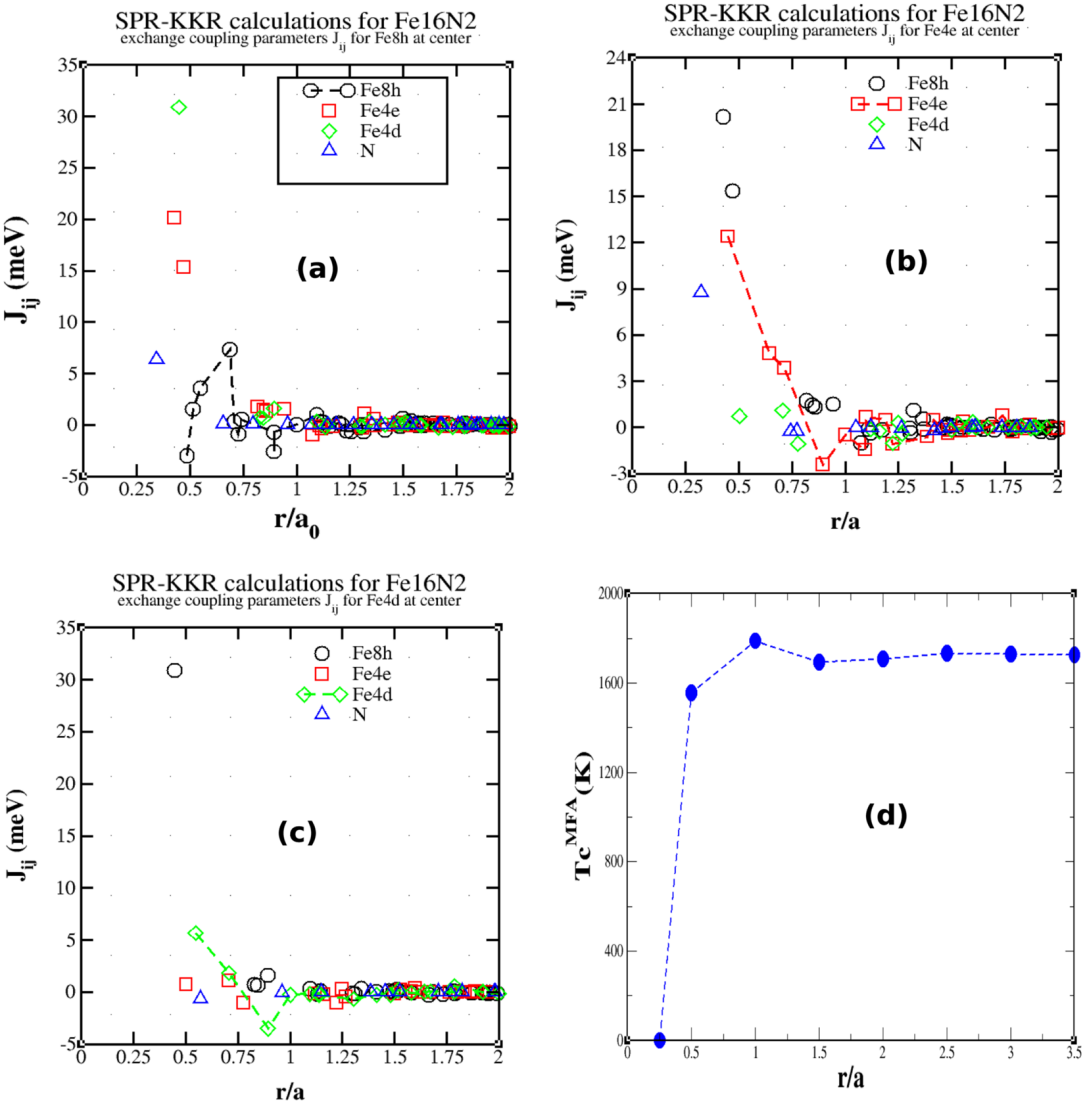
Exchange parameters for (**a**) Fe8h, (**b**) Fe4e, and (**c**) Fe4d as centers. (**d**) The calculated Curie temperatures for various values of the radius of the sphere, within which all interactions were considered.

In Fig. 1, we show the calculated exchange constants that were obtained via the SPR-KKR method using the PBE functional. Although the trend of the results is similar to that of the results that were obtained by Ke *et al*.^11^ via the TB-LMTO method under the local density approximation (LDA) and the GW method, there are substantial differences. The largest among all interactions is between the 8h-4d sites (30.89 meV) at a distance of 2.54 Å, as shown in Fig. 1(a)), while for Ke *et al*. it was between the 8h-4e sites. The next-strongest interaction from our calculation is between the 8h-4e sites (20.15 meV) (Fig. 1(a)) at a distance of 2.43 Å between two octahedral Fe atoms. The third-strongest interaction (15.34 meV) is between the octahedral 8h and 4e sites at a distance of 2.67 Å. Due to structural considerations, this interaction should be mediated through a 90° Fe8h-N-Fe4e superexchange interaction. Among the inter-sublattice interactions, the 4d-4e interaction is the weakest (0.74 meV) (Fig. 1(b)). According to Fig. 1(a), the 8h-8h interactions (between Fe atoms that are residing at different Wyckoff positions) are antiferromagnetic (−2.97 meV) at the smallest distance (2.76 Å). These interactions may correspond to a 90° Fe8h-N-Fe8h superexchange interaction. The strongest Fe8h-Fe8h interaction is ferromagnetic (3.58 meV) at 3.9 Å and it is due to a 180° Fe8h-N-Fe8h superexchange interaction. All the strong magnetic interactions that involve either 8h-4d or 8h-4e sites are nearly along the [111]-direction, similar to bcc Fe; however, due to the structural distortion (in the case of Fe_16_N_2_), these lattice vectors are slightly deviated. For example, the vector that connects the 8h and 4d sites is [0.25, 0.24, 0.27]. The interactions between the 4d and 4e sites are nearly along the [011]-direction. For bcc Fe, the most prominent interaction along the [011]-direction is anti-ferromagnetic, as calculated by Pajda *et al*.^14^; however, in the present case, it is still ferromagnetic, although very weak. This suggests that Fe_16_N_2_ is a much stronger ferromagnet than bcc Fe.

Among the intra-sublattice interactions (between the Fe atoms that are at the same Wyckoff positions), the 4e-4e interaction is the strongest (12.39 meV), which corresponds to the interaction between two Fe4e atoms in two neighboring octahedra. The next-strongest 4e-4e interaction is between two epical Fe atoms in the Fe-N octahedra and, therefore, is mediated by a 180° superexchange interaction. The strongest 4d-4d interaction is 5.3 meV at a pair distance of 3.1 Å and, therefore, is almost negligible.

In Fig. 1(d), we show the variation of the mean-field Curie temperature as a function of the cluster radius, which is denoted as |**r**|. Even for a small radius of approximately |**r**| = 0.5 Å, T_*C*_ is much higher than the experimental value (813 K); hence, the mean-field picture does not explain the T_*C*_ value of this material. MFA overestimates T_*C*_ due to neglect of the thermal fluctuations of magnetization. However, in the present case, MFA overestimates T_*C*_ by almost 50%, which is large. Such overestimation of T_*C*_ of Fe_16_N_2_ by MFA was also observed by Ke *et al*.; however, they attributed this to the lack of a proper experimental measurement of T_*C*_. Such consistent huge overestimation of T_*C*_ by MFA reflects a low-dimensional behavior of the spin system compared to a pure 3D one. The origin of such quasi-2D-like behavior could be the poor connectivity between spins that differ in terms of direction. There is almost no exchange between spins at the 4e and 4d sites and a very weak 4d-4d interaction is observed in Fig. 1.

**Figure 2:**
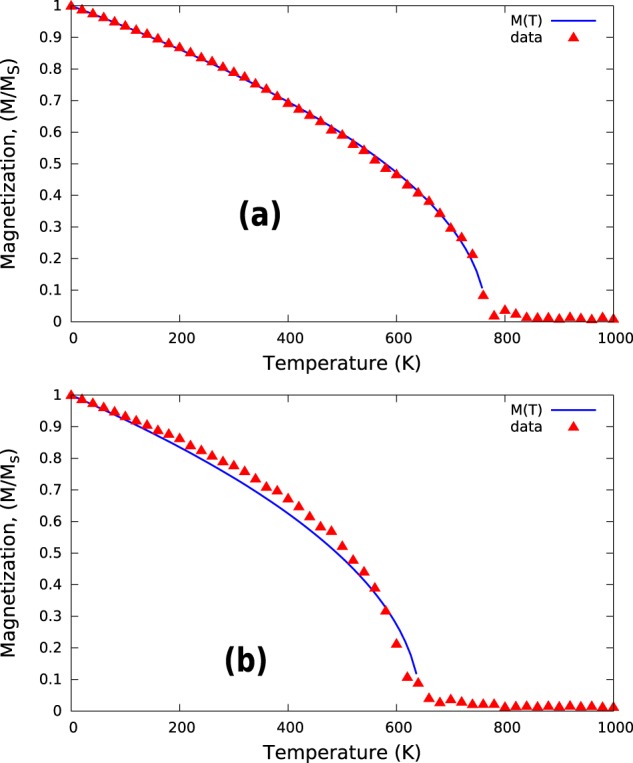
Monte Carlo simulation of temperature-dependent magnetization. The top panel (a) corresponds to pure Fe_16_N_2_ and the bottom panel (b) to V-doped Fe_16_N_2_.

To obtain more accurate results, we performed MC simulation using the metropolis method^15^. In Fig. 2, we present our results for the MC simulations. To obtain the value of T_*C*_, we fit the temperature-dependent magnetization data to the function $M(T)={(1-\frac{T}{{T}_{C}})}^{\beta }$, which yields a T_*C*_ value of approximately 765 K according to Fig. 2(a), which is much closer to the experimental value compared to the MFA results that were obtained by us and those that were obtained by Ke *et al*. For comparison, we also performed MC simulation for the V-doped Fe_16_N_2_. We demonstrated in our previous study^10^ that Fe_16_N_2_ can be stabilized by doping V at site 8h; hence, we performed the MC calculations with exchange constants that were obtained via the SPR-KKR method by replacing one Fe-8h with V. Figure 2(b) shows the obtained results. The T_*C*_ value that was obtained after fitting is approximately 640 K, which is less than that for bulk Fe_16_N_2_, but still above room temperature.

## Understanding the *giant magnetic* Moment

We will discuss the origin of the giant magnetic moment in this section. As already discussed in the introduction, the experimental findings about such moments are not consistent. Therefore, the reliability of such reports is uncertain. In the following, we analyze the possibility of a *giant* moment being present in this system. The following two factors that might influence the saturation magnetization are discussed here: (1) extrinsic effects such as strain, which we analyze from the fixed magnetic moment calculations, and (2) intrinsic effects such as the existence of localized or semi-localized d-states within the octahedral region and exchange interactions that favor the high-spin configuration of the 3d-states, which we investigate based on the magnetic susceptibility and the nature of the exchange interactions.

### Structural sensitivity and magnetism: constrained magnetic calculations

To elucidate the relation between the structure and magnetism, we performed constrained moment calculation with the PBE exchange correlation function. The magnetic moment of the cell was fixed such that each Fe site had a magnetic moment of approximately 3 *μ*_*B*_. Under this constraint, we relaxed the cell geometry completely, which yielded lattice parameters a = b = 5.84 Å and c = 6.34 Å. The c/a ratio remains close 1.10 (as in the unconstrained case); hence, the large magnetic moment does not result in any additional structural anisotropy, such as additional tetragonality. The space-group (No. 139) remains almost the same after the structural optimization with giant moments. The only difference appears in the position parameters with z_4*e*_ = 0.3 (0.293 for unconstrained case) and x_8*h*_ = 0.240 (0.242 in the unconstrained case). This results in a slight increase in the distance between N and Fe at the 8h and 4e sites. Next, we performed a calculation that involves the optimization of positions while the volume of the cell is fixed to the volume that was obtained via the constrained magnetic calculation that is described above. We allowed the atomic spin moments to change with the electronic steps in this case. This resulted a magnetic moment of 2.52 *μ*_*B*_ per Fe atom, which is slightly larger than the values that are reported in Table 1. This calculation suggests a weak correlation between the structure and the magnetic moment in Fe_16_N_2_. Therefore, it is highly unlikely for such large moments to appear due to strain effects that arise from substrates or via other means. Therefore, the origin of giant magnetic moment should be related to intrinsic physics such as partial localization of d-electrons in the Fe-N octahedra or the nature of the exchange interaction promoting the maximization of the local spins through Hund’s coupling. The partial localization of d-electrons in Fe_16_N_2_ was demonstrated by Ji *et al*.^9^; however, they demonstrated it in terms of the difference in charge density between inside and outside the Fe_6_N octahedra.

**Table 1:** Lattice constants and magnetic moments in three types of GGA calculations.

GGA	a (Å)	c (Å)	m_8*h*_ (*μ*_*B*_)	m_4*e*_ (*μ*_*B*_)	m_4*d*_ (*μ*_*B*_)	x_8*h*_	z_4*e*_
PBE	5.68	6.22	2.36	2.18	2.82	0.242	0.293
PBE-sol	5.75	6.28	2.40	2.17	2.84	0.242	0.294
Revised PBE	5.75	6.28	2.42	2.22	2.86	0.243	0.294

### Intrinsic effects: partial localization of d-electrons in Fe_6_N octahedra and high spin states being favored by exchange interactions

**Figure 3:**
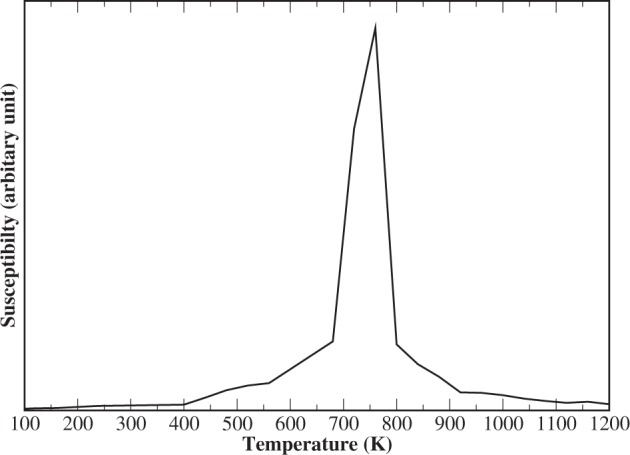
Magnetic susceptibility of bulk Fe_16_N_2_ that was obtained from MC simulations.

In Fig. 3, we show the calculated magnetic susceptibility as a function of the temperature. The susceptibility shows a sharp peak at T = T_*C*_, which indicates the presence of localized states in this material. For itinerant systems, the magnetic susceptibility usually shows a broad peak below T = T_*C*_. Therefore, it is important to investigate the possibility of localized electronic states that contribute to the magnetic moments.

**Figure 4:**
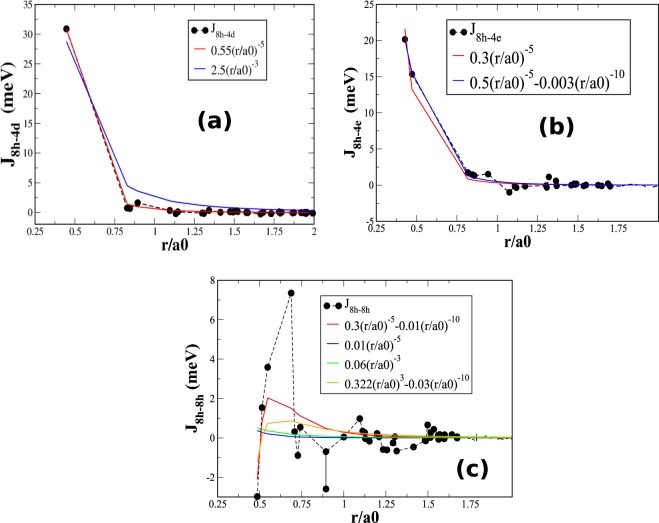
Numerically obtained magnetic exchange interactions that involve the octahedral 8h site: (**a**) 8h-4d, (**b**) 8h-4e, and (**c**) 8h-8h interactions. These values are fitted using various models, as discussed in Section IV.

In Fig. 4, we analyze the magnetic exchange interactions (obtained via SPR-KKR) that involve 8h sites within the framework of the tight-binding model. Within the tight-binding model, the intersite hopping integral, which is denoted as *t*_*dd*_, between the Fe sites that involve only d-orbitals can be expressed as ${t}_{dd} \sim {(r/a\mathrm{0)}}^{-5}$^16^. First, we discuss the strongest among all the interactions: the interaction between the Fe-3d states at the 8h and 4e sites. In Fig. 4(a), for the 8h-4d interactions, we fit the exchange constants with two itinerant-type interactions: the double-exchange and RKKY interactions. Typically, the double-exchange interaction is directly proportional to *t*_*dd*_, namely, *r*/*a*0^−5^, while the RKKY interaction is proportional to (*r*/*a*0)^−3^. According to the figure, *J*_8*h*−4*d*_ accords extremely well with the double-exchange type. Due to its double-exchange nature, the *J*_8*h*−4*d*_ interaction is strongly ferromagnetic. However, for the 8h-4e interactions (Fig. 4(b)), a linear combination of double-exchange and superexchange interactions of the form *J* = *α*(*r*/*a*0)^−5^ + *β*(*r*/*a*0)^−10^ accords well (as the superexchange interaction has ${t}_{dd}^{2}$-dependence), where *α* and *β* are the fitting parameters. Finally, for 8h-8h interactions, we fit the exchange constants with four types of interactions: (1) linear combinations of double-exchange and superexchange interactions (red curve), (2) double-exchange interactions (blue curve), (3) RKKY interactions (green curve), and (4) combinations of RKKY and superexchange interactions (yellow curve). According to the figure, the behavior of the data most strongly resembles an interaction that is represented by case (4), namely, a combination of double-exchange and superexchange interactions. The substantial tendency of the exchange interactions within the octahedra toward superexchange interactions suggests partial weak localization of electron states at 8h-sites.

The strongest magnetic interaction in this material is between 8h and 4d sites (30.89 eV per pair) and it is of the double-exchange type. This was first predicted by Sakuma *et al*.^17^. Our conclusion regarding the high moment is as follows: Due to the presence of N, there is a moderate correlation effect in the octahedral region that localizes partially the d-states within this region (especially the 8h states), along with a strong tendency of double-exchange interactions to prefer high-spin states^18^.

**Figure 5:**
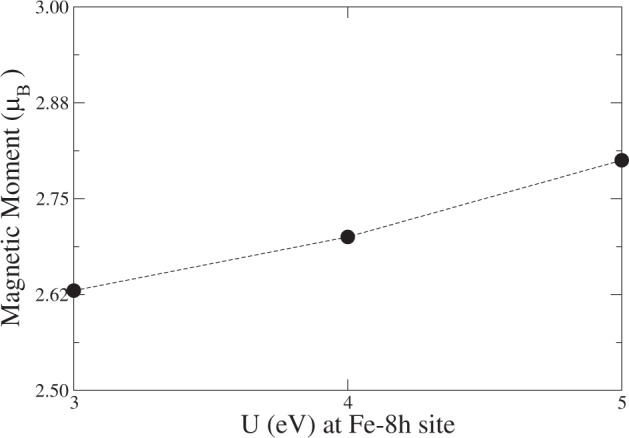
Magetic moments per Fe for various U values at the 8h site.

To determine the extent to which the partial localization of the octahedral d-states may affect the magnetic behavior or the saturation moment of Fe_16_N_2_, we computed the magnetic moments per unit cell via the DFT + U method^19^ with various values of U, as shown in Fig. 5. Switching on the localization effect at a moderate level (3–5 eV) gives rise to a magnetic moment that is as high as $\sim 2.8{\mu }_{B}$. For typical Fe-based insulating systems, the typical choice for U is approximately 6 eV. As Fe_16_N_2_ is a metal, the U value should be smaller than 6 eV.

## Fe_16_N_2_ as a Spin Injector

**Figure 6:**
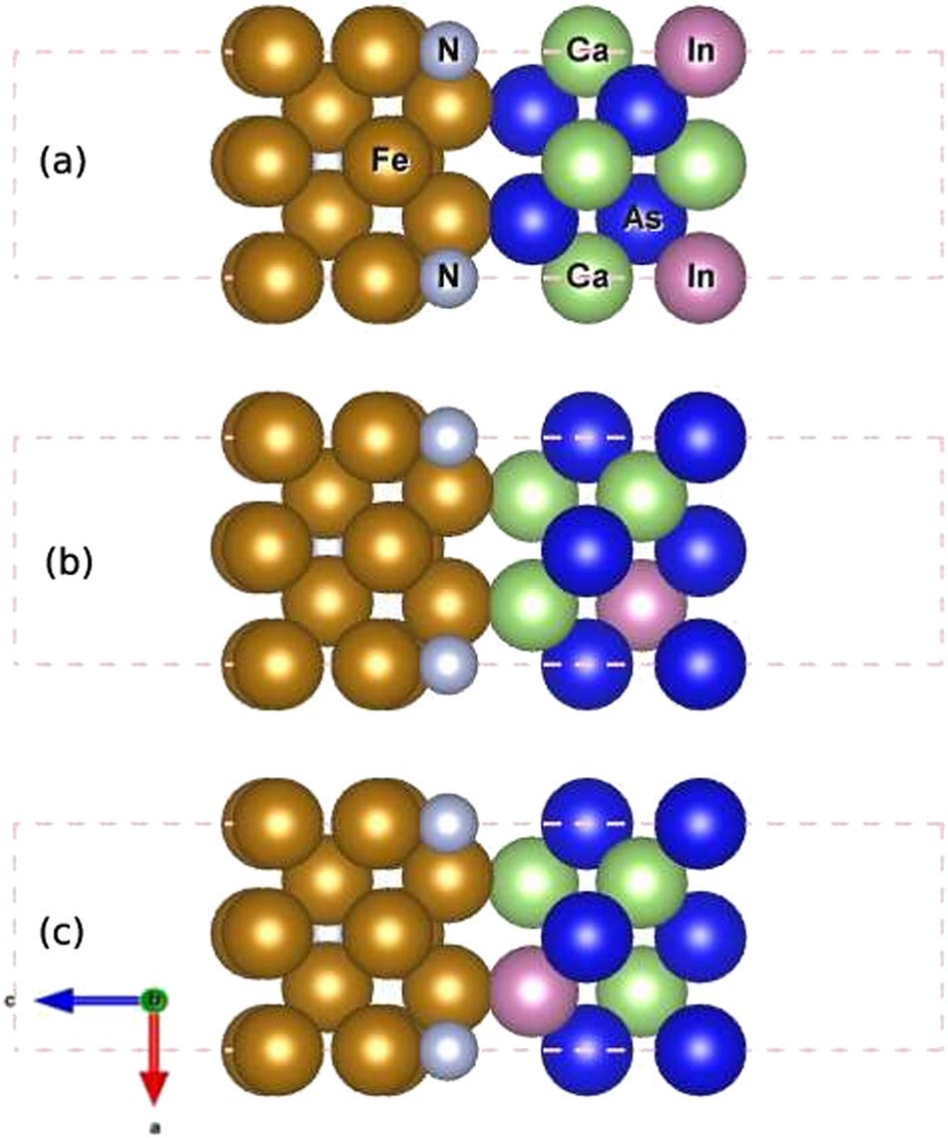
Fe_16_N_2_(001)/InGaAs(001) thin films with 4 monolayers of metal and semiconductor for three configurations.

The bulk spin polarization that we obtained for the PBE-optimized Fe_16_N_2_ is approximately 60%, which is large compared to the elemental metallic ferromagnets, such as Fe (45%), Co (42%) and Ni (27%)^20^. Such large spin polarization falls in between the half-metallic Heusler alloys (~100%), which were proposed to be the optimal spin-injectors for the semiconductors^21^, and the elemental ferromagnets. However, until now, the Heusler alloys were not highly successful spin injectors due to their large lattice mismatch with the semiconductors^22,23^. Fe_16_N_2_ can be easily grown on III–V semiconductors such as GaAs or InGaAs (historically, the giant moment was observed in epitaxially grown single crystals of Fe_16_N_2_ on either InGaAs (001) or GaAs(001) film). As Fe_16_N_2_ has both large spin polarization and satisfactory lattice matching with semiconductors, we examined its application as a spin injector as follows. We performed calculations with 4 monolayers (ML) of Fe_16_N_2_ (001) on 4 ML of InGaAs (001) and 8 ML of InGaAs (001). The lattice parameters of bulk InGaAs were obtained. The optimized structure was used to construct the metal/semiconductor (001) interfaces, as shown in Fig. 6. The magnetic moments per Fe for configurations (a), (b) and (c) are 2.48 *μ*_*B*_, 2.51 *μ*_*B*_ and 2.52 *μ*_*B*_, respectively. The magnitude of the moment depends on the nature of the interface. The magnetic moment is higher for interfaces that contain either Ga or In. These moments are not *giant*; however, they are comparable to the maximum value of the saturation moment that is predicted by the Slater-Pauling curve^24^. Then, we investigate the possibility of spin injection from the ferromagnet (FM) to the semiconductor (SC) in such systems. As an example, we have computed the layer-resolved spin polarization, namely,

$${P}_{L}=\frac{{D}_{L}^{\uparrow }({E}_{F})-{D}_{L}^{\downarrow }({E}_{F})}{{D}_{L}^{\uparrow }({E}_{F})+{D}_{L}^{\downarrow }({E}_{F})},$$

where L is the layer index and ${D}_{L}^{\uparrow (\downarrow )}({E}_{F})$ is the density of states at the Fermi energy for the majority (minority) of spin carriers for interface (b) in Fig. 6. The results are shown inFig. 7, which presents the layer-dependent magnetic moments in the top panels. On the Fe_16_N_2_ side, all four layers have moments that are close to 2.5 *μ*_*B*_ (the average is approximately 2.51 *μ*_*B*_), while the spin polarization (bottom panel) decreases as we approach the interface. However, on the semiconductor side (the 5^*th*^ to 8^*th*^ layers), the spin polarization is high and is non-negligible, even up to the last layer. The average spin injection efficiency over all four layers is approximately 30%, which is higher than (almost double) what is observed for Fe/GaAs heterostructures^25^. This implies a long spin diffusion length in Fe_16_N_2_/InGaAs systems. The spin polarization changes sign across the interface; hence, the spin imbalance in the semiconductor side is due to the minority spin.

**Figure 7:**
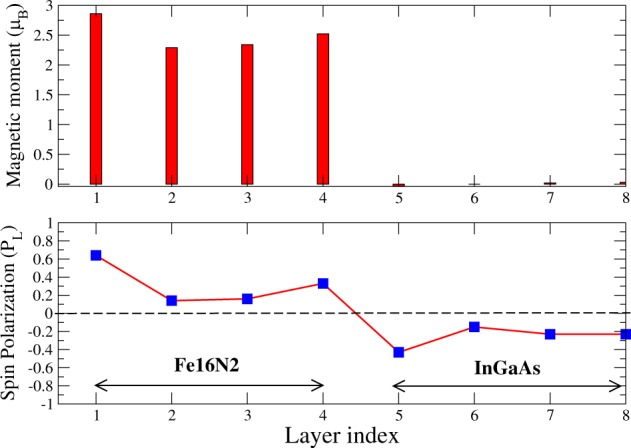
(Top panel) Layer-resolved magnetic moments per atom for the Fe_16_N_2_(001)/InGaAs(001) thin-film configuration (**b**) of Fig. 6. (Bottom panel) The corresponding layer-resolved spin polarization.

Fe_16_N_2_ seems to be a highly effective spin injector for III–V semiconducting systems such as InGaAs. As shown in our previous study^10^, small V doping stabilizes Fe_16_N_2_; therefore, its interface with III–V semiconductors might be important for spin-optoelectronic or spintronic^26,27^ applications.

## Conclusions

We have studied the exchange interactions and magnetic properties of Fe_16_N_2_ and V-doped Fe_16_N_2_ via first-principles-based methods. The magnetism and T_*C*_ are dominated by the exchange interactions between 8h-4d and 8h-4e sites. By fitting the exchange parameters that were obtained via the first-principles-based method to various theoretical models of exchange interactions, we demonstrate that in addition to the double-exchange interaction, a substantial role is played by the superexchange interaction. Although the double-exchange interaction being the strongest favors a high-spin state, the presence of the superexchange interaction among the Fe sites within the Fe_6_N octahedral region indicates the existence of localized states, which may also contribute to the occurrence of a giant moment in this material. In addition, due to its high spin polarization and fantastic lattice matching with the III–V semiconductors, Fe_16_N_2_ can also find important spintronic applications in addition to the permanent magnet application.

## Computational Method

The electronic structure calculations were performed via first-principles methods within the framework of density functional theory (DFT) with the Perdew-Burke-Ernzerhof exchange correlation energy functional^28^ based on a generalized gradient approximation. We used a projector-augmented wave method as implemented in the Vienna *ab-initio* simulation package (*VASP*)^29^. Kohn-Sham wave functions of the valence electrons were expanded in a plane-wave basis with an energy cut-off of 450 eV. The augmentation charge cut-off was set to 627.1 eV. The Brillouin zone sampling was conducted using a Monkhorst Pack grid of 7 × 7 × 7 k-points. Ionic relaxation was performed using the conjugate-gradient method until forces were reduced to within 0.01 eV/Angstrom. The calculated structural parameters, such as the lattice constant, positions, and magnetic moments, are employed to obtain the exchange parameters using Korringa-Kohn-Rostoker (KKR) multiple-scattering theory. We have used the Munich *SPR-KKR* package^30^ to obtain the exchange parameters via a real-space approach that was formulated by Liechtenstein *et al*.^31^, which maps the total energy of the system to an effective Hamiltonian that is expressed as

1 $$ {\mathcal H} =-\sum _{i\ne j}\,{J}_{ij}{{\bf{e}}}_{i}{{\bf{e}}}_{j}$$

where **e**_*i*_ represents the vector of the local magnetic moment of the i^*th*^ site and *J*_*ij*_ denotes the exchange constant between sites *i* and *j*. The angular momentum expansion of the basis functions was calculated up to l = 3. The k-space integration was performed using a grid of 280 k-points in the irreducible part of the Brillouin zone. We have used 30 complex energy points to perform the integration over the Green’s function. The Curie temperature within MFA was calculated by solving the following coupled equations:

2 $$\langle {{\bf{e}}}^{\alpha }\rangle =\frac{2}{3{K}_{B}T}\sum _{\beta }\,{J}_{0}^{\alpha \beta }\langle {{\bf{e}}}^{\beta }\rangle $$

where 〈**e**^*α*^〉 is the average z-component of the magnetic moment in sublattice *α* and ${J}_{0}^{\alpha \beta }={\sum }_{{\rm{r}}}\,{J}_{0{\rm{r}}}^{\alpha \beta }$. The summation in **r** is performed up to |**r**|/*a* = 3.0. To obtain an accurate value of T_*C*_, we conducted Monte Carlo simulations with the *VAMPIRE* atomistic spin dynamic program^32^. We used 67200 atoms with periodic boundary conditions. The DFT-calculated magnetic moments (from *VASP*) and exchange constants (from *SPR-KKR*) were used as inputs. The Hamiltonian of the system contained the following exchange and anisotropy terms:

3 $$ {\mathcal H} =-\sum _{ij}\,{J}_{ij}{{\bf{e}}}_{i}{{\bf{e}}}_{j}-{K}_{u}\sum _{i}{({{\bf{e}}}_{i}\cdot {\bf{e}})}^{2}$$

where **e** is the unit vector along the direction of the easy axis and K_*u*_ is the uniaxial anisotropy constant. The values of K_*u*_ for pure and doped Fe_16_N_2_ were obtained from our previous paper^10^.
